# Characterization of a new *oda3* allele, *oda3-6*, defective in assembly of the outer dynein arm-docking complex in *Chlamydomonas reinhardtii*

**DOI:** 10.1371/journal.pone.0173842

**Published:** 2017-03-14

**Authors:** Jason M. Brown, Matthew Mosley, Daniela Montes-Berrueta, Yuqing Hou, Fan Yang, Chasity Scarbrough, George B. Witman, Maureen Wirschell

**Affiliations:** 1 Department of Biology, Salem State University, Salem, Massachusetts, United States of America; 2 University of Mississippi Medical Center, Department of Biochemistry, Jackson, Mississippi, United States of America; 3 University of Massachusetts Medical School, Department of Cell and Developmental Biology, Worcester, Massachusetts, United States of America; East Carolina University Brody School of Medicine, UNITED STATES

## Abstract

We have used an insertional mutagenesis approach to generate new *C*. *reinhardtii* motility mutants. Of 56 mutants isolated, one is a new allele at the *ODA3* locus, called *oda3-6*. Similar to the previously characterized *oda3* alleles, *oda3-6* has a slow-jerky swimming phenotype and reduced swimming speed. The *oda3-6* mutant fails to assemble the outer dynein arm motor and outer dynein arm—docking complex (ODA-DC) in the ciliary axoneme due to an insertion in the 5’ end of the *DCC1* gene, which encodes the DC1 subunit of the ODA-DC. Transformation of *oda3-6* with the wild-type *DCC1* gene rescues the mutant swimming phenotype and restores assembly of the ODA-DC and the outer dynein arm in the cilium. This is the first *oda3* mutant to be characterized at the molecular level and is likely to be very useful for further analysis of DC1 function.

## Introduction

Cilia and flagella (terms here used interchangeably) are highly conserved organelles with both motile and sensory functions. Defects in assembly or function of cilia lead to a class of diseases known as ciliopathies—pleiotropic pathologies that affect development and adult human organ function [1–10]. Studies of cilia in *C*. *reinhardtii*, and the dynein motors that drive their movement, have been instrumental in the identification of numerous ciliopathy genes [11].

*C*. *reinhardtii* provides an excellent model for studies of dynein subunit composition, assembly and regulatory mechanisms. The ciliary dynein motors power ciliary movement by generating the forces to power normal ciliary bending and beating [12]. There are two main classes of ciliary (or axonemal) dynein motors: the outer dynein arms (ODA) and the inner dynein arms (IDA). The ODAs provide most of the power for ciliary movement and contribute mainly to ciliary beat frequency. The IDAs, of which there are several sub-types, contribute to ciliary waveform (the size and shape of the ciliary bend).

**Fig 1:**
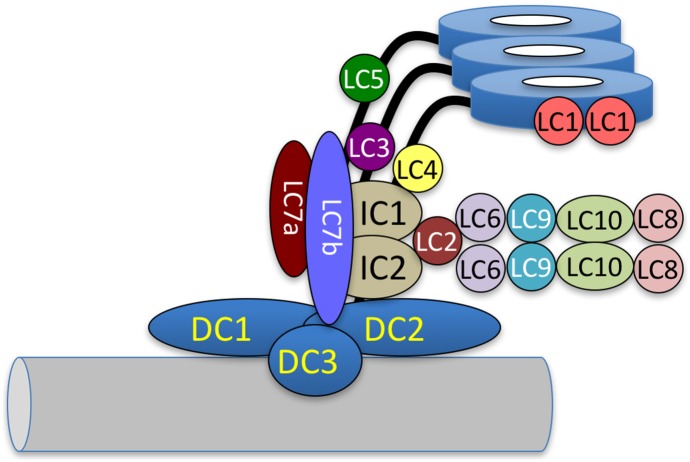
Subunit composition of the *C*. *reinhardtii* ODA and ODA-DC. The ODA is a multi-subunit complex consisting of three heavy chain (HC), two intermediate chain (IC), and a series of light chain subunits. The ODA is anchored to the outer doublet microtubule (grey tube) via the ODA-DC, a heterotrimeric complex containing DC1, DC2 and DC3.

The subunit composition of the ODA in *C*. *reinhardtii* has been well defined. The ODA consists of three heavy chain motor subunits (α, β and γ), two intermediate chains (IC1 and IC2), and a series of smaller light chain subunits (LC1-6, LC7a, LC7b, LC8-10) (Fig 1). The ODA motors are positioned at 24-nm intervals along the length of the ciliary axoneme. ODAs are closely associated with a second complex, called the ODA-docking complex, or ODA-DC, which also has a 24-nm periodicity along the axoneme [13–15]. The ODA-DC is a heterotrimeric complex consisting of two coiled-coil proteins (DC1 and DC2) and a redox-sensitive calcium-binding subunit (DC3) [16–18]. (Note that the nomenclature for the ODA-DC genes and subunits is *DCC1*, *DCC2* and *DLE3* for gene names; *oda3*, *oda1* and *oda14* for mutant alleles; DC1, DC2 and DC3 for proteins [19]). It is required to anchor the ODA to the ciliary outer doublet microtubules [13, 15] but is not required for the 24-nm periodicity of the ODA [20].

Genetic mutations in genes encoding components of the ciliary dynein motors, components required for dynein assembly, and components of other ciliary structures linked to motility cause Primary Ciliary Dyskinesia (PCD—one of the founding ciliopathy diseases) [5, 21, 22]. Moreover, recent data demonstrates that alcohol exposure, akin to chronic alcohol consumption, alters the activity of the ODA resulting in an “acquired” ciliopathy called Alcohol-Induced Ciliary Dysfunction [23]. Given that there are genetic and acquired ciliopathies involving the ODA, it is important to understand the molecular mechanisms for ODA assembly and regulation of ODA function. In order to further our understanding of ODA assembly, we isolated insertional mutant strains in *C*. *reinhardtii* displaying defects in ciliary motility. Here, we describe one mutant strain, JB09H1, which harbors a deletion in the *DCC1* gene encoded at the *ODA3* locus. This mutation represents a new *oda3*-mutant null allele, which we designate as *oda3-6*.

## Results

*Identification of JB09H1 as an* oda *mutant*: The wild-type strain, g1, was transformed with the *AphVII* selectable marker cassette [24] to produce individual insertional mutant transformants that are resistant to hygromycin. Isolated transformants were analyzed by light microscopy for the presence/absence of flagella and for motility defects resulting in altered swimming. The transformant termed JB09H1 displayed a classic Oda-motility phenotype: slow-jerky movement with greatly reduced forward swimming speed. This swimming phenotype is consistent with other *oda* mutants that fail to assemble the ODA in the axoneme [25].

**Fig 2:**
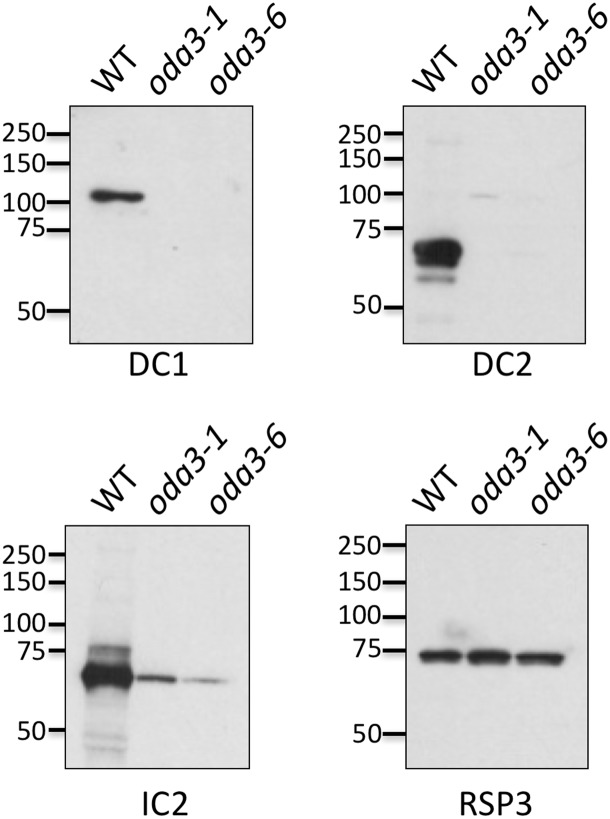
The *oda3-6* mutant is defective in assembly of the ODA-DC. Isolated axonemes from wild type (the g1 parent strain), *oda3-1* and *oda3-6* were analyzed by western blot. Antibodies to IC2 demonstrate that the ODA is greatly reduced in *oda3-1* as expected as well as in the newly isolated JB09H1 strain (*oda3-6*). Anti-DC1 and -DC2 antibodies demonstrate that, as in *oda3-1*, the ODA-DC is missing in *oda3-6* axonemes. Antibodies to RSP3 serve as a loading control.

To determine the assembly status of the ODA in the JB09H1 transformant, we isolated axonemes and analyzed them by immunoblot (Fig 2). Using antibodies to the ODA intermediate chain IC2, we find that assembly of the ODAs is greatly reduced in the JB09H1 mutant. Previous studies have demonstrated that the ODA-DC is required for assembly of the ODA into the axoneme [16–18]. Using antibodies to DC1 and DC2, we find that both ODA-DC proteins are missing in the JB09H1 mutant. These results demonstrate that the JB09H1 motility phenotype is caused by loss of the ODA-DC and consequently the ODA in the axoneme. These results are consistent with the phenotype associated with mutations in either the *DCC1* [17] or the *DCC2* gene [18].

**Fig 3:**
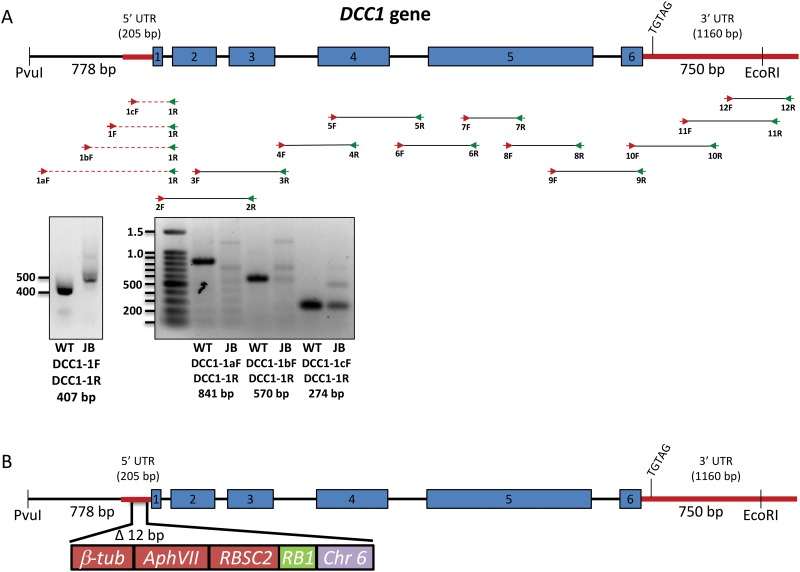
PCR defines the mutation in the 5’ end of the *DCC1* gene. **(A)** PCR primers (see Table 1) were used to screen the *DCC1* gene in *oda3-6* DNA compared to wild-type DNA. Primers in the 5’ end of the *DCC1* gene fail to amplify *DCC1* sequences. **(B)** PCR with primers from the hygromycin-resistance cassette and *DCC1* further defined the insertional mutation in the *DCC1* gene. The transforming hygromycin cassette inserted into the *DCC1* 5’ UTR resulting in a deletion of 12 bp of *DCC1* sequence. Sequencing of PCR products demonstrates that the 5’ junction site reads into the β-tubulin promotor of the hygromycin cassette. The 3’ junction extends from the *RBSC2* 3’ UTR of the hygromycin cassette into ~500–600 bp of the *H*. *sapiens RB1* gene followed by 383-bp of chromosome 6. These results demonstrate that JB09H1 is defective in the *DCC1* gene and is a new mutant allele at the *ODA3* locus, which we designate as *oda3-6*.

*JB09H1 is an allele at the* ODA3 *locus*: To determine the molecular defect in JB09H1, we performed a PCR screen to analyze the *DCC1* and *DCC2* genes for defects (Fig 3 and Table 1). We did not identify any defects in the *DCC2* gene (data not shown). In contrast, we identified a region in the 5’ end of the *DCC1* gene, located on chromosome 17, that generates a larger PCR product in the JB09H1 mutant than in wild type (primers DCC1-1F and DCC1-1R; Fig 3A). Sequence analyses of the JB09H1 PCR product demonstrates that the DNA is primed at both ends by the reverse primer, which amplifies non-*DCC1* sequences (data not shown). Three additional PCR primers were designed in this region. Two (DCC1-1aF and DCC1-1bF) amplify *DCC1* sequences of the expected size from wild-type DNA, but fail to amplify DCC1 sequences from the mutant DNA. A third primer (DCC1-1cF) amplifies a non-specific band of the wild-type size from the mutant DNA. This indicates that this region of the *DCC1* gene is mutated and that the JB09H1 strain harbors a new *oda3* allele. Previous studies have isolated five *oda3* alleles [17, 25]. Therefore, the JB09H1 allele is termed *oda3-6*.

**Table 1:** DCC1 and DCC2 primers used in this study.

DCC1-1F	CATGGGTGCAAAGTTCTAAGG	DCC2-1F	ATTGTCTTGTTATGCCTATCGTC
DCC1-1R	GAGCTCGGGCGATCTCTAC	DCC2-1R	ACCTTACCTTTGCTTGTCCAG
DCC1-2F	TCTGCGGACAAAGGAGGAGTTG	DCC2-2F	GCATTATAAGCAAGTGACATAAAGG
DCC1-2R	GAGGCATCCGTGGTGTCGTC	DCC2-2R	TGTTCCGGGTGGTGGTTAG
DCC1-3F	CAAGGGGCCCGGTATGGACAC	DCC2-3F	CGCGCTTATTTGCAGATTGTGTTG
DCC1-3R	AGCTGGCGCGACTGGAAGAAACAAC	DCC2-3R	GGTTGTGAGCTGTCGCCATTCC
DCC1-4F	AGGGTGCGGGTGGGGTGTTGTTTC	DCC2-4F	ACGTGAAGTACAACCAGTCCATTAC
DCC1-4R	GCTGCTTCTGCTGCCGCTTCTTG	DCC2-4R	GTTCGCAACGGTAGGCACAG
DCC1-5F	TCACGGCGCACTTCAACGAGATG	DCC2-5F	GCAATTCGCACACGCAAACTG
DCC1-5R	AAGAAGGGGGCGGGGAAGGCTATC	DCC2-5R	GTCGATGTCCTCAATACCTGTGG
DCC1-6F	GGGATGGCGCCAGAGGGGGTAG	DCC2-6F	CCTGCTGGTGGCGAGTTTG
DCC1-6R	ATCTCCTCCAGGGCAATCATCAGCC	DCC2-6R	GCTGACGTATGAATGCGGATG
DCC1-7F	ACATGGAGGGGGCGAAGGGCAAG	DCC2-7F	TCTTCGGGTGCTGGGACTGTC
DCC1-7R	CTCGGGCAGCTCAGGGAAGAAGTGC	DCC2-7R	AGCAAGAATGAGCAAACCGTGTAAG
DCC1-8F	ACAAGCCGTCGTCGCCTCTG	DCC2-8F	CAGGCCATGGTCGTGAAAGAGAGG
DCC1-8R	TCTTCCCCTTCTTCCGCTTGATG	DCC2-8R	ATCAGTACAGCACACGCCCACAAAAC
DCC1-9F	TGGGGCGGTGGGGCAGAG	DCC2-9F	TGGGGAGGTGGCAGAGTTGATGTG
DCC1-9R	CGCGCATTACACCTTGATGGCG	DCC2-9R	GGCGGTGACAAGCGGTGATGAG
DCC1-10F	TCGTGGACCGTGACTACATCAAGC		
DCC1-10R	CAACTCAATCACCCCTCCCACTGC		
DCC1-11-F	ACGCTCGTGACTGGATTTACATGCCC		
DCC1-11R	CCACACCACGCGCCTGCCTTTTG		
DCC1-12F	AGGGCTGGAACGCTGAGATAG		
DCC1-12R	TGCAACCGACATTAGCCATTC		
DCC1-1aF	CCGCCTTCGTCTTAAACTTG		
DCC1-1bF	CTGGGCTCTTTTGCTAGACG		
DCC1-1cF	CCGCGCTAACGAAGCAAAG		
DCC1-DMR2	CAGCTTCTTCTGCCAGTCCA		

**Fig 4:**
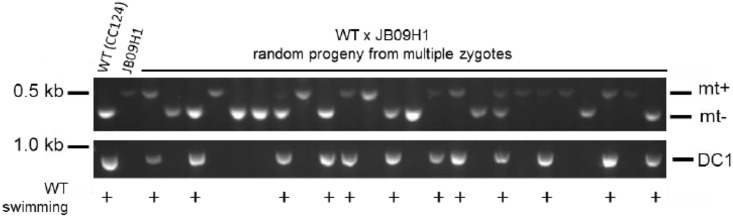
Analysis by PCR demonstrates that the *DCC1* gene deletion is tightly linked to the Oda-mutant phenotype. The *oda3-6* (JB09H1) strain was backcrossed to a wild-type strain and the resulting progeny were analyzed for motility (+ WT swimming) and presence or absence of the *DCC1* gene defect. All progeny that display wild-type motility also successfully amplify the 5’ end of the *DCC1* gene (PCR performed with primers DCC1-AF and DCC1-1R to generate an 841-bp product) demonstrating they have an intact *DCC1* gene. All progeny that have the Oda-mutant phenotype lack the PCR product demonstrating they contain the mutant *DCC1* gene.

To further define the mutation in *oda3-6*, we performed PCR with primers specific to the *AphVII* cassette (primers DP4, DP3, DP2, DPS, UP3, UP2, UP1, and UPS) [26] combined with DCC1-specific primers (DCC1-1aF, DCC1-1R, DCC1-DMR2; Table 1) to amplify sequences covering this region. We determined that the hygromycin-resistance cassette inserted into the *DCC1* 5’ UTR. We defined the *DCC1*/hygromycin-resistance cassette junctions and determined that the insertional event resulted in the deletion of 12 bp of the *DCC1* 5’ UTR located 55 bp upstream of the start codon (Fig 3B). Sequencing of PCR products revealed that the 5’ junction site contains sequences from the 5’ UTR of the *DCC1* gene immediately adjacent to the β-tubulin promotor of the hygromycin-resistance cassette. The 3’ junction between *DCC1* and the hygromycin-resistance cassette contains 383-bp from chromosome 6 (bp 3904945–3905327). In between the chromosome 6 sequences and the *RBSC2* 3’ UTR of the hygromycin-resistance cassette are ~500–600 bp of relatively poor sequence quality that does not match any *C*. *reinhardtii* sequence. This sequence is at least 88% percent identical to the *Homo sapiens RB1* gene over more than 500 bp and was likely introduced as contaminating DNA during the transformation procedure. These results are consistent with the high frequency of complicated insertions observed by us and described by others [27]. Genetic backcrosses of *oda3-6* to a wild-type strain, CC124, demonstrate that the deletion in the *DCC1* gene co-segregates with the Oda-mutant swimming phenotype (Fig 4).

**Fig 5:**
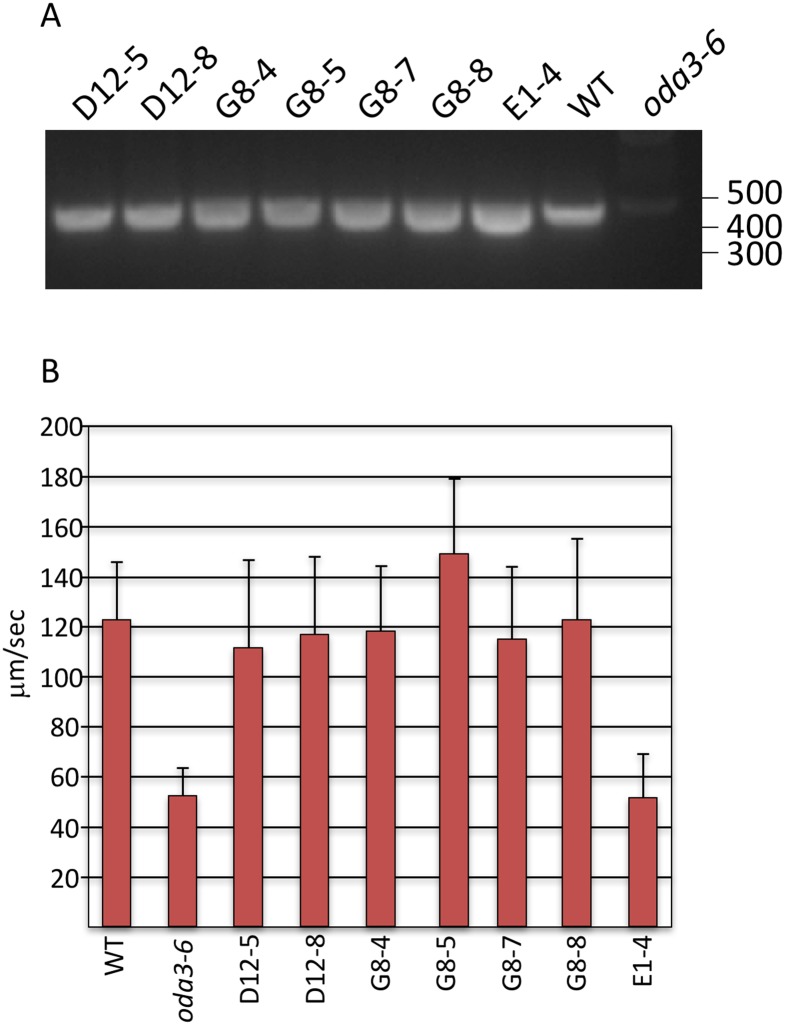
Transformation of *oda3-6* with the wild-type *DCC1* gene restores the missing *DCC1* sequences and rescues ODA motility. **(A)** Genomic DNA from the g1 parental strain (WT), *oda3-6* and the selected transformants were analyzed by PCR for the presence or absence of the 5’ end of the *DCC1* gene using primers DCC1-1F and DCC1-1R. All transformant strains amplified a product as does WT demonstrating that the *DCC1* transgene successfully integrated into the genome of the transformants. As shown in Fig 3, the *oda3-6* strain is missing this part of the *DCC1* gene and does not amplify a product. **(B)** Swimming speed measurements demonstrate that the D- (D12-5 and D12-8) and G-transformants (G8-4, G8-5 and G8-8) have recovered wild-type swimming velocities (compare to WT). In contrast, the E1-4 transformant does not display rescued motility and swims at speeds comparable to *oda3-6*.

*The wild-type* DCC1 *gene rescues the* oda3-6 *motility phenotype*: To demonstrate that the motility defect in *oda3-6* is caused by mutation in the *DCC1* gene, we transformed the *oda3-6* strain with a plasmid containing a genomic fragment encompassing the *DCC1* gene. Previous studies have rescued *oda3* alleles using a large ~10-kb genomic fragment [17]. Here, we have excised a smaller region encompassing the *DCC1* gene and tested its ability to rescue the *oda3-6* mutant phenotype. The *oda3-6* strain was transformed with the smaller *DCC1* transgene construct. Transformants were selected using the paromomycin selectable marker and subsequently screened for motility and integration of the *DCC1* transgene (Fig 5). To verify that the *DCC1* transgene had successfully integrated, we analyzed genomic DNA from the wild-type parent strain, the *oda3-6* strain and the motile transformants (Fig 5A). All three transformant series (D, G and E) amplify the 5’ end of the *DCC1* gene, which is deleted in *oda3-6*.

We quantitated swimming velocities to confirm that the *DCC1* transgene fully rescues the *oda3-6* motility defect (Fig 5B). The average swimming speed of the wild-type parent strain is 123 ± 20 μm/sec, whereas the *oda3-6* mutant swims significantly slower at 53 ± 11 μm/sec (p = <0.001). Transformants D12-5 and D12-8 (siblings), and G8-4, G8-5, G8-7 and G8-8 (siblings) swim at speeds comparable to the wild-type strain and are significantly faster than the *oda3-6* mutant (p = <0.001). In one G- transformant, G8-5, the average swimming speed was actually greater (145 ± 34 μm/sec; p = <0.001) than the wild-type parent strain. In contrast, strain E1-4 displays the paromomycin resistance phenotype and contains the *DCC1* transgene (Fig 5A), yet does not show rescued motility. Altogether, two independent transformants (D and G series) displayed rescued motility (Fig 5B).

**Fig 6:**
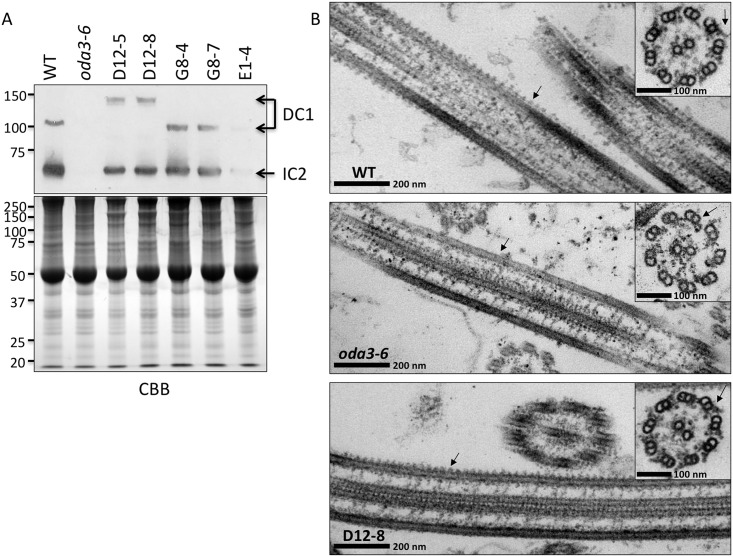
Transformation of *oda3-6* with the wild-type *DCC1* gene rescues ODA assembly. **(A)** Top panel: Antibodies to DC1 demonstrate that the ODA-DC is assembled in wild type (WT), D- and G-transformant axonemes. As a result, assembly of the ODA is restored in these strains (as shown by antibodies detecting the IC2 subunit of the ODA). Notably, the DC1 protein in the D-transformants migrates as a larger protein, which indicates that this integration event results in expression of a chimeric protein. In contrast, the E1-4 transformant is not rescued for ODA and ODA-DC assembly (as shown by much less DC1 or IC2 detected in the axonemes), which is consistent with the failure to restore wild-type swimming in this strain**. Bottom panel: a gel of** the same samples was stained with Coomassie brilliant blue (CBB) as a loading control. **(B)** Electron microscopy demonstrates that the ODAs (arrows in top and bottom panels) assemble with normal periodicity on all eight outer doublet microtubules in wild type (WT) and the D-transformant (D12-8). As expected, the *oda3-6* strain lacks the ODAs (arrows in middle panels).

*The* DCC1 *transformants are rescued for ODA assembly*: To verify that the integrated *DCC1* transgene rescues the motility phenotype by restoring ODA assembly, we performed immunoblot analyses on isolated axonemes (Fig 6A). As expected, axonemes from the wild-type parent strain contain both the ODA and ODA-DC as represented by IC2 and DC1 respectively. The *oda3-6* mutant lacks both the ODA and ODA-DC. Both the D and G transformants show both IC2 and DC1 in the axoneme demonstrating that the *DCC1* transgene is expressed and restores ODA and ODA-DC assembly. Interestingly, in the D transformants, DC1 migrates in SDS-PAGE more slowly with a relative mobility of ~150 kDa, indicating that the transgene integration created a chimeric protein. The G transformants show a slightly smaller DC1 protein relative to wild-type axonemes. Strain E1-4 has greatly reduced levels of IC2 and DC1, consistent with the failure to rescue motility that is observed in this transformant.

To further verify the rescue of ODA assembly in the transformants, we analyzed isolated axonemes by electron microscopy. The parent strain shows normal assembly and periodicity of ODAs on eight outer doublet microtubules (Fig 6B**, WT**), whereas the *oda3-6* strain lacks ODAs (Fig 6B**, *oda3-6***). Electron microscopy of one of the D transformants (Fig 6B**, D12-8**) demonstrates that ODA assembly and periodicity are fully restored despite the greater length of DC1 in these strains.

## Discussion

Here, we describe the isolation and characterization of a new *oda3* null allele designated *oda3-6*. This mutant harbors an insertion of the transforming hygromycin cassette along with a small fragment from chromosome 6 and a short segment of *Homo sapiens* DNA within the 5’ UTR of the *DCC1* gene (Fig 3). The *DCC1* gene encodes the DC1 subunit of the ODA-DC, a complex required for ODA assembly. Failure to assemble the ODA results in reduced ciliary beating and a slow-jerky motility, a phenotype displayed by the *oda3-6* strain (Fig 5).

We successfully rescued the *oda3-6* mutant phenotype using a *DCC1* transgene coupled with direct selection by resistance to paromomycin. Notably, the *DCC1* transgene, which is smaller than that previously used to rescue an *oda3* mutant [17], also rescues the original *oda3-1* mutant strain (data not shown). This *DCC1* transgene contains the entire 205 bp 5’UTR that is specified in the *Chlamydomonas* Genome Database, Phytozome v5.5, along with 750 bp of the 1160-bp 3’ UTR. The 3’ UTR in the *DCC1* transgene includes the only predicted polyA signal consensus sequence (TGTAG) in the entire 1160-bp 3’ UTR (Fig 3A). Thus, we predict this transgene contains all the required elements for proper expression of the DC1 protein and rescue of the *oda3-6* mutant phenotype. The *DCC1* transgene successfully integrated into three independently isolated transformants (D, G and E). However, the E1-4 strain does not show rescued motility, ODA assembly or ODA-DC assembly as shown by western blotting (Figs 5 and 6). Thus, for unknown reasons, even though the *DCC1* transgene integrated in E1-4, the assembly of DC1, in the axoneme, is inefficient in this strain.

Both the D and G- transformants express abnormally sized DC1 proteins. The D-transformants express a larger DC1 protein (Fig 6A) indicating that the integration event in these cells results in expression of a chimeric protein. While the specific nature of this chimeric protein is unknown, the fusion does not appear to affect ODA-DC /ODA assembly or function as we observe wild-type motility in these cells. The G-transformant axonemes contain a slightly smaller DC1 protein suggesting the integration event in these cells occurred such that the *DCC1* gene is truncated, likely at the 3’ end. Like the D-transformants, the G-transformants show wild-type swimming velocities and normal ODA-DC/ODA assembly. These abberent integration events likely reflect the use of uncut plasmid DNA for the transformations.

Recent studies have revealed that the ODA-DC is not required for the periodicity of the ODA’s [20]; rather, the 24-nm periodicity is an intrinsic feature of the ODAs. Structural studies indicate that the ODA-DC is a flexible structure that serves to enhance the binding of ODAs to the doublet microtubules. The flexibility of the ODA-DC may accommodate the movements of the ODA motor domains during ciliary movement. Consistent with these models, the larger DC1 protein in the D-transformants does not alter the periodic assembly of ODA motors (Fig 6B).

The generation of new mutants that are defective in ODA assembly or function is important for furthering our understanding of ODA assembly mechanisms and how mutations affecting the ODA contribute to ciliopathies like PCD. The *oda3-6* allele is the first *oda3* allele to be characterized at the molecular level. The *oda3-1* and *-2* alleles were generated by UV irradiation [25] and thus may be point mutations that are hypomorphic alleles or susceptible to true reversion. The *oda3-4* and *-5* mutants were generated by insertional mutagenesis using a technique that often results in large deletions that can disrupt several genes at the insertion site [28–31]. Because *oda3-6* is a null mutant involving a small deletion in the *DCC1* 5’ UTR, it is likely to be very useful for further studies using genetically engineered versions of *DCC1* to investigate ODA-DC function and precise location in the axoneme.

## Materials and methods

*Strains and culture conditions*: *C*. *reinhardtii* strains used in this study include: g1 (*nit1*, *NIT2*, *agg1*, *mt+*); CC124 (137c, *mt-*), CC2232 (*oda3-1*, *mt+*), JB09H1 (*oda3-6*, mt+, this study), D12-5 (*oda3-6*::*ODA3*, mt+, this study), D12-8 (clonal isolate of D12-5), G8-4 (*oda3-6*::*ODA3*, mt+, this study), G8-5, G8-7 and G8-8 (clonal isolates of G8-4), and E1-4 (*oda3-6*::*ODA3*, mt+, this study). “CC” strains were obtained from the *Chlamydomonas* Stock Center (St. Paul, MN).

Cells were cultured on Tris-acetate-phosphate (TAP) agar plates in an incubator (Percival model I66LLC8) with a 14 hr: 10 hr light/dark cycle and 65% relative humidity. For analysis of swimming cells and isolation of flagella, cells were cultured in TAP liquid media with aeration.

Genetic crosses were performed according to standard methods [32]. JB09H1 (mt+) was crossed to wild-type strain, CC124 (mt-). Random progenies were selected and scored for motility by light microscopy and for the absence *DCC1* gene sequences and mating type by PCR.

*Insertional mutagenesis and transformation of the oda3-6 strain*: Strain g1 was transformed with a restriction fragment containing the *AphVII* selectable marker for hygromycin resistance [24]. Transformants were selected on TAP plates supplemented with 10 μg/ml hygromycin B (Sigma-Aldrich, St, Louis, USA). Hygromycin-resistant colonies were picked into liquid cultures and those displaying motility phenotypes of interest were kept.

Clone pAKEXX18#3 contains a ~10-kb genomic fragment encompassing the wild-type *DCC1* gene [17]. This clone was digested with EcoR1 and PvuI to release a 4.5-kb fragment containing the *DCC1* gene. The EcoR1-PvuI fragment was treated with Klenow polymerase and the blunted fragment cloned into the Sma1 site of vector pSE280 to make plasmid pCS3.3. The *AphVIII* cassette for paromomycin resistance was PCR-amplified from plasmid PSI103 [33] and cloned into the PCR Script Amp vector (Stratagene, La Jolla, CA) to make plasmid PMM1 (Table 2). A 1.8-kb EcoRV-Not1 restriction fragment from PMM1, containing the paromomycin- resistance cassette, was cloned into plasmid pCS3.3 to make plasmid pFY8.1. The *oda3-6* strain was transformed with plasmid pFY8.1 and transformants selected on TAP plates supplemented with 7.5 μg/ml paromomycin [33]. Paromomycin-resistant transformants were analyzed by light microscopy to score the swimming phenotype.

**Table 2:** Paromomycin cassette primers used in this study.

PMM-cassette F	GACGGCGGGGAGCTCGCT
PMM-cassette R	GGTACCCGCTTCAAATACGCC

*Analyses of swimming speed*: Cells were cultured in liquid TAP for 1–2 days and cell movement was digitally recorded as described [23]. The data presented is the mean swimming speed and standard deviation of ~30 cells. The statistical significance was determined by the *t*-test method using Microsoft Excel.

*PCR*: Regions spanning the *DCC1* and *DCC2* genes were amplified using the primers listed in Table 1. PCR reactions were analyzed on 1% agarose gels and imaged using a digital imaging system (BioRad, Hercules, CA). Mating type was verified by PCR using primers listed in Table 3. The PCRs to delineate the mutation in *oda3-6* were performed with UP and DP primers specific for the hygromycin-resistance cassette [26] combined with DCC1 forward and reverse primers (DCC1-1aF, DCC1-1R, and DCC1-DMR2; Table 1).

**Table 3:** Mating type PCR primers used in this study.

Mt+	*fus3*	TCCAACGCATAGCCATCAAC
Mt+	*fus4*	TGTTTGCTAGGGGTGCAATG
Mt-	*mid1*	ACCGGTGTTTACCGTCGAGT
Mt-	*mid2*	CCTTTCTGTAGGGCCACCTG

*Isolation of axonemes*: Ciliary axonemes were isolated as described with slight modifications [34]. Cells were grown in TAP liquid media and concentrated by centrifugation. The wash step in 10 mM Hepes, pH 7.4 was omitted. Deflagellation was induced by exposure to Dibucaine, the flagella collected and demembranated with 1% NP-40 alternative (EMD Millipore, Billerica, MA) in HMDEKP buffer (30 mM Hepes pH 7.4, 5 mM MgSO4, 1 mM DTT, 0.5 mM EDTA, 25 mM KCl, 1 mM PMSF and additional protease inhibitors (Complete protease inhibitors, Roche, Indianapolis, IN). Axonemes were collected by centrifugation and fixed for SDS-PAGE by addition of 5X sample buffer (50 mM Tris pH 8.0, 160 mM DTT, 5 mM EDTA, 50% sucrose, 5% SDS).

*SDS-PAGE and Immunoblotting*: Axonemal proteins were separated on 7% SDS-PAGE gels and then transferred to nitrocellulose (BioRad, Hercules, CA). The blots were probed with antibodies to DC1 (1:1,000; provided by Ken-ichi Wakabayashi, Tokyo Institute of Technology, Japan), DC2 (1:1,000; provided by Ken-ichi Wakabayashi, Tokyo Institute of Technology, Japan), IC2 (1:10,000; Sigma Aldrich, St. Louis, MO) and RSP3 (1:10,000) [35]. Antibody reactivity was visualized with HRP-conjugated secondary antibodies (BioRad, Hercules, CA) and a chemiluminescent substrate (GE Healthcare, Pittsburgh, PA). The same protein samples were stained with coomassie brilliant blue (CBB) for protein loading controls.

*Electron Microscopy*: Ciliary axonemes were isolated as described above with minor modifications. Cells were grown in liquid M media (medium I [36] altered to have 0.0022 M KH_2_PO_4_ and 0.00171 M K_2_HPO_4_) aerated with air supplemented with 5% CO_2_ to mid-log phase. Cells were washed once in 10 mM Hepes, pH 7.4 before deflagellation. The flagella were demembranated with 0.1% NP-40 alternative in HMDEK buffer (30 mM Hepes pH 7.4, 5 mM MgSO4, 1 mM DTT, 0.5 mM EDTA, 25 mM KCl). Axonemes were collected by centrifugation, briefly washed with HMDEK buffer, and then prepared for electron microscopy as described by Hoops (1995) with modifications [37]. Briefly, the pelleted axonemes were fixed for 1 hour and 15 minutes in fixative (2% glutaraldehyde, 1% tannic acid in 100 mM sodium cacodylate buffer). Axonemes were then washed three times with 100 mM cacodylate buffer, post-fixed in 1% osmium tetroxide in 75 mM cacodylate buffer, washed twice in 50 mM cacodylate buffer, washed three times in water, and then stained overnight in 1% uranyl acetate. Samples were dehydrated through a graded ethanol series, embedded in Polybed 812/Araldite 502, and sectioned at 50–70 nm. Sections were stained with uranyl acetate and lead citrate and viewed in a Phillips CM10 microscope.
